# Variables influencing telemedicine utilization via telephone appointments among rural patients

**DOI:** 10.1186/s12913-024-12122-5

**Published:** 2025-01-02

**Authors:** Eliseo García, Benjamin C. Vincent, Shivani Thakur, Ashish Thakur, Fatima Zabiba, Sandhini Agarwal, Jasmin Dominguez Cervantes, Ahmed Zabiba

**Affiliations:** 1 Valley Vein Health Center, 840 Delbon Ave, Turlock, CA 95382 USA; 2 Davis School of Medicine, University of California, 4610 X St, Sacramento, CA 95817 USA; 3 Georgetown University School of Medicine, 3900 Reservoir Rd NW, Washington, DC 20007 USA; 4 Stanford University, 450 Jane Stanford Way, Stanford, CA 94305 USA; 5 College of Osteopathic Medicine, California Health Science University, 2500 Alluvial Ave, Clovis, CA 93611 USA

**Keywords:** Patient preference, Vascular surgery, Varicose veins, Venous medicine, Clinical competence

## Abstract

**Objective:**

To evaluate patient preferences when utilizing telemedicine.

**Methods:**

A 5-point Likert scale questionnaire was completed by 153 patients at a rural clinic via a convenience sampling method. The survey contained 21 statements encompassing provider confidence, patient-physician rapport, and accessibility variables. Patient responses for those who attended (*n* = 120) or canceled/rescheduled (*n* = 33) telemedicine encounters were analyzed using paired difference t-tests and t-tests of correlations between different groups of variables. An ad-hoc method patterned after the least significant differences was applied to the analysis of variance results to evaluate patient-valued variables.

**Results:**

Patients surveyed included 82.4% female (*n* = 127) and 17.6% male (*n* = 26); 69.3% of them were Hispanic/Latino (*n* = 106). When reviewing the patient opinion statements, our data suggested a belief that telemedicine encounters were as good as in-person visits (*n *= 118, x̄ = 4.932) and that such encounters provided them with the confidence to proceed with future, in-person vein treatments (*n* = 117, x̄ = 4.744). Additionally, patients expressed feeling as though their personal information was safe (*n* = 117, x̄ = 4.897).

**Conclusion:**

Rural patients indicated a preference for flexible encounters and for providers who strive to build trust and rapport when utilizing telemedicine.

**Supplementary Information:**

The online version contains supplementary material available at 10.1186/s12913-024-12122-5.

## Background

Telemedicine is the use of communication technology to provide healthcare services remotely [1]. With the rise in technological advancements, the sharing of medical information over large distances has advanced through telegraphs, telephones, and the internet. In modern times, healthcare providers can deliver care directly to patients in the comfort of their own homes via live chat services that allow real-time one-on-one communication [2]. Through various telemedicine methods, including telephone calls and electronic health records, patients can schedule appointments, have access to their medical history, and communicate with their providers [3]. This can be both time and cost-effective for the patient, as it resolves the barrier of physically going to the provider’s office to receive information and care that could otherwise be communicated virtually.

One month after the Centers for Disease Control and Prevention declared COVID-19 to be a pandemic in April 2020, the number of telemedicine insurance claims in the United States (US) increased from 0.15% to 13% (an 86-fold difference as compared to April 2019 [4]), as healthcare departments transitioned from in-person to remote encounters [5–7] due to concerns regarding SARS-CoV-2. Prior to the pandemic, however, telemedicine utilization was already on the rise, increasing in US hospitals by 41% from 2010–2017, while also being employed by 61% of healthcare institutions across the nation [8, 9]. This could partly be explained by the modality’s affordability (vs. in-person encounters) and convenience, amongst other benefits [9]. However, there are concerns regarding the efficacy of some forms of telemedicine. For instance, the direct-to-consumer version of the modality does not necessarily rely on a patient’s typical clinician and it may lack appropriate medical tests and equipment, thereby minimizing the data available for making diagnoses and aptly prescribing medications [10]. Similarly affecting providers’ ability to serve them well, patients have also been noted to downplay the signs and symptoms of their ailment during telephone consultations [11].

Nonetheless, as telemedicine’s quality continues to be refined and its utilization expands, it is prudent to begin identifying patient-valued interface traits to maximize its scope of use. This is particularly relevant for the field of rural vascular surgery, as a recent study has indicated that the modality is acceptable for the follow-up care of chronic venous disease (CVD) [12]. Generally speaking, telemedicine services can deliver high-quality care for patients with venous disease in a safe and coordinated manner, with varicose vein patients having demonstrated high satisfaction with telemedicine over the traditional healthcare delivery model [13]. With telemedicine’s affordability, this could help address the CVD treatment costs of the approximately 40% of Americans who are affected by the condition, [14] which are estimated to be around $150 million to $3 billion [15, 16]. Additionally, telemedicine can improve the quality of care and access in rural areas, but limited evidence is available on how telemedicine is perceived by these patients [17]. Although previous studies have focused on telemedicine’s efficacy in specific fields and on patient satisfaction, to the best of our knowledge, few studies have evaluated which traditional healthcare aspects rural patients would appreciate within the interface. Moreover, systematic review studies found no substantial differences between telephone and video telehealth appointments, especially with regard to clinical effectiveness and patient satisfaction [18].

During the early weeks of the expansion of telemedicine, telephone visits were the common use of the modality [17]. As time progressed the rise of video visits increased, however, this has become a barrier to underserved populations [17, 19]. Patients using video visits have been more likely to be White, enrolled in commercial insurance, and living in areas with higher income and broadband access. Patients who are older than 65 years, Black, Hispanic, and from areas with low broadband access are less likely to use video visits [17, 19]. Video visits compared to telephone visits require a complex setup and broadband internet access, which may present barriers for older adults, racial/ethnic minorities, and those with limited English proficiency (LEP) [17, 19].

Accordingly, Valley Vein Health Center (VVHC) created a patient survey to gain insight into CVD rural patients’ views on telemedicine through telephone visits. This paper aims to highlight variables, which rural patients with CVD rated favorably when employing telemedicine services.

## Methods

The study was conducted from January to February 2021 at VVHC, a rural outpatient clinic with seven separate locations serving Central California. All surveys were conducted in accordance with relevant guidelines and regulations approved by the Valley Vein Health Center Ethics and Institutional Review Board (IRB) Committee. A convenience sampling method, using a predetermined survey, was used with an informed consent process that was a voluntary, opt-in consent-by-completion approach for all subjects. The survey was developed through a comprehensive review of primary research and review articles from international sources, including data from the United States, the United Kingdom, and Iran, which had previously identified variables contributing to in-person care hesitancy. The 21 survey items were carefully constructed based on these established variables. Specifically, 18 of the items were derived from the most prevalent in-person care hesitancy variables, which had been previously grouped in the literature into three major categories: provider confidence, patient-physician rapport, and treatment accessibility. These categorizations were supported by existing research findings and were used as the basis for structuring the survey. The remaining three items were designed to assess patient opinions specific to their individual healthcare encounters. Overall, these predetermined survey questions were intended to mitigate the challenge of receiving incomplete questionnaires (i.e. having surveys returned with many unanswered items), which may have arisen from free-response questions.

The survey was tested and reviewed by 15 staff members and 30 patients for clarity and validity before a final set of questions was approved. Additionally, a statistician, a patient advocate, and VVHC’s two clinicians reviewed the questionnaire for appropriateness. The finalized survey had two versions available in English and Spanish: one for patients who completed their appointment and one for patients who canceled/delayed it. The latter survey had statements that generally opposed those found in the “completed appointment” survey. For the ranking of individual survey items, a five-point Likert rating scale was employed: a rating of five indicated that a statement was favorable or true, whereas a rating of one meant that a statement was unimportant or false (please see Appendix 1).

Using a convenience sampling method with a voluntary, opt-in consent by-completion approach, 153 distinct patients who had attended (*n* = 120) or canceled/rescheduled (*n* = 33) an over-the-phone telemedicine appointment completed the survey. Patients completed questionnaires over the phone either before or after their scheduled consultation with their provider by the research assistants at VVHC, as well as by a researcher (EG). For the completion of the cancellation/rescheduling surveys, the research assistants and researcher (EG) contacted patients who had delayed a healthcare encounter at VVHC over the past year, with respect to the study’s conclusion date. All surveys were conducted over the phone and patients were explained the reasons for the study and what it hoped to answer, in addition to its benefits.

Paired difference t-tests were utilized to compare the general variable categories to one another. A t-test of the correlation between each pair of categories (i.e. confidence vs. rapport, confidence vs. accessibility, and rapport vs. accessibility) was employed to determine whether an observed correlation was significantly different from zero. Additionally, least significant difference (LSD) tests modeled after Bonferroni's LSD in Analysis of Variance (ANOVA) were applied to the survey data to determine statistical significance when comparing the favorability of individual categories to one another.

## Results

Patients surveyed at VVHC were 82.4% female (*n* = 127) and 17.6% male (*n* = 26); 69.3% of them were Hispanic/Latino (*n* = 106), 22.2% were White (non-Hispanic/Latino) (*n *= 34), 3.3% were Indian/Pakistani/Punjabi (*n* = 5), 2% were Black/African American (*n* = 3), 2% were Asian (*n* = 3), and 1.2% were American Indian/Alaska Native (*n* = 2). 24.9% of these individuals (*n* = 38) completed an English version of the questionnaire, while 75.1% of them (*n* = 115) completed a Spanish version. The following proportions of patients belonged to the respective age groups: 3.9% were 18–30 (*n* = 6), 15.7% were 31–40 (*n* = 24), 21.6% were 41–50 (*n* = 33), 26.8% were 51–60 (*n* = 41), 21.6% were 61–70 (*n* = 33), 8.4% were 71–80 (*n* = 13), and 2% were 81 + (*n* = 3). Overall, 6.5% (*n* = 10) were new to our telemedicine services, while 93.5% (*n* = 143) were returning telemedicine patients.

When seeking to identify the top patient preferences, LSD tests revealed variables 1–14 (see Table 1) to be significantly different from variables 15–18, although there were no clear distinctions within each group of variables (1–14 and 15–18). There are some negligible differences among the variables at the edges of 1–14, but these individual statements’ statistical overlap essentially leaves them as a single large group with items that only differ in non-significant or barely significant ways. For the favorability of these traits to be statistically different from one another, the difference between the means of the individual items being compared (e.g. 1 vs. 2, 1 vs. 3, or 2 vs. 3, etc.) must be greater than the LSD of 0.242 for variables 1–14; the aforementioned means refer to the overall average Likert rating that a distinct statement received. Similarly, for any item to be statistically distinguishable from traits 15–18, the difference between the means of the individual statements being compared must be greater than the LSD of 0.403. Nonetheless, based on the mean Likert ratings of patient responses, the top favored variables are: services with providers who are kind and helpful (*n* = 119, x̄ = 4.966), visits with physicians who are considered knowledgeable (*n* = 119, x̄ = 4.958), the implementation of technology that acknowledges COVID-19 safety concerns (*n* = 119, x̄ = 4.950), the provision of care by staff who are considered knowledgeable (*n* = 120, x̄ = 4.917), and appointments with physicians and staff who are perceived as trustworthy (*n* = 119, x̄ = 4.916). The mean Likert values for all variables, as well as examples of significant values calculated from the difference between these means, are summarized in Table 1.

**Table 1:**
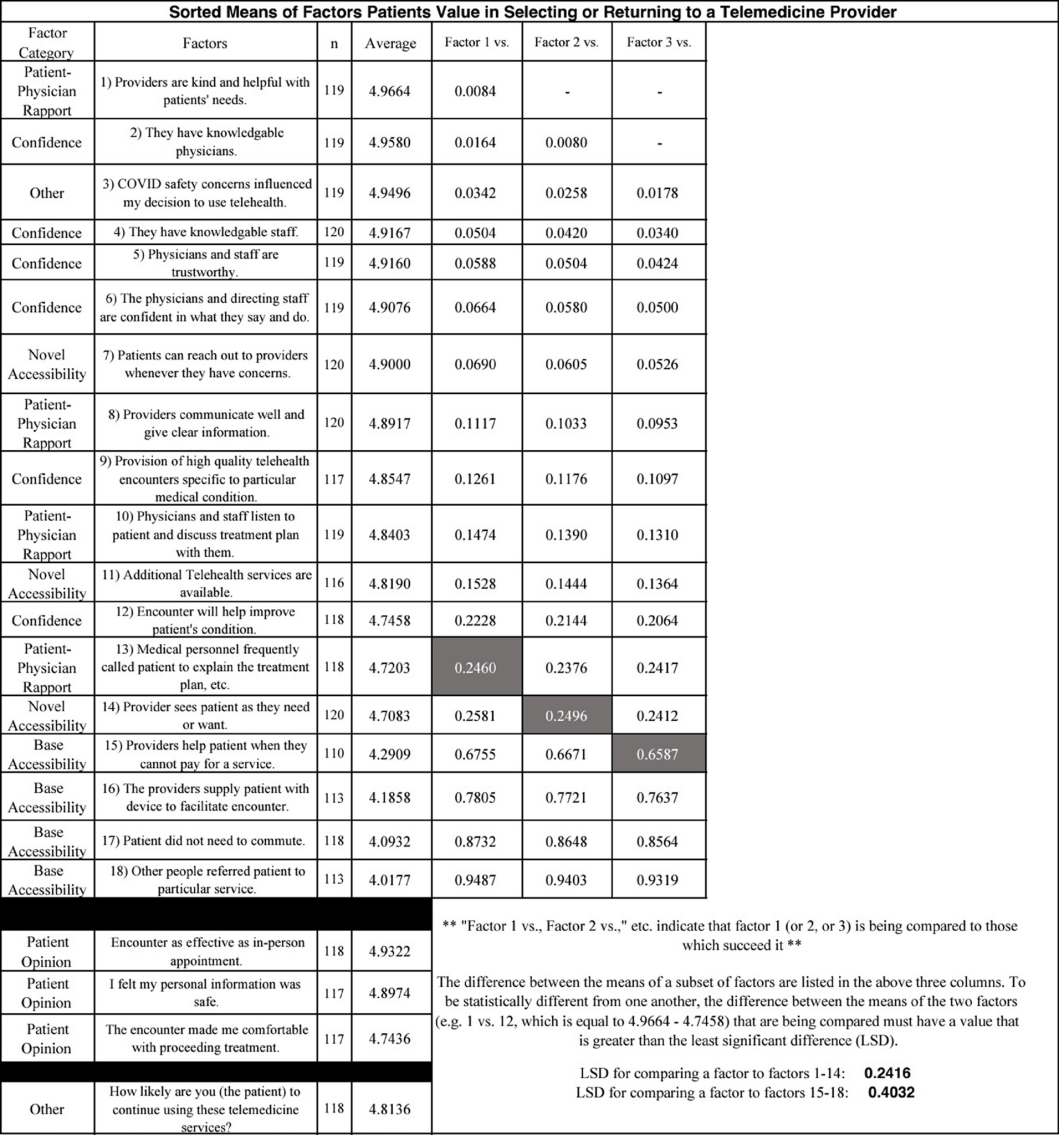
This study rated variables valued by patients who completed telemedicine visits versus those who did not complete telemedicine visits

When reviewing the patient opinion statements, our data suggested a belief that telemedicine encounters were as good as in-person visits (*n* = 118, x̄ = 4.932) and that such encounters provided them with the confidence to proceed with future, in-person vein treatments (*n* = 117, x̄ = 4.744). Additionally, patients expressed feeling as though their personal information was safe (*n* = 117, x̄ = 4.897). Overall, completed surveys revealed that telemedicine is a promising modality for phlebology consultations (*n* = 118, x̄ = 4.814), with only three respondents indicating that they will not likely use such services in the future.

In reviewing the mean Likert ratings, patients indicated some accessibility variables to be more important than others, which were termed as “novel,” since these variables have not been widely noted by previous studies to be valued by patients who utilize telemedicine. Those variables that were less appreciated were termed “base,” as they generally coincide with variables that were prevalent in past studies. Novel accessibility variables include ease in contacting providers and flexibility in encounter availability. Base accessibility variables include patient referrals, appointments not requiring a commute, the provision of devices to facilitate telemedicine interaction (if necessary), and financial assistance for the service. These and all other survey items are individually listed in Table 1. 

Additionally, paired difference t-tests suggest that patients generally placed less value on accessibility variable statements when compared to patient-physician rapport and confidence variable statements. When comparing the novel and base accessibility variable statements to the confidence variable statements, patients indicated the latter to be more valued than either of the former variable statements (x̄_d_ = 0.076, SD = 0.399, t = 2.082, *p* = 0.039 and x̄_d_ = 0.739, SD = 0.924, t = 8.763, *p* = 1.563E-14, respectively; x̄_d_: mean of the differences of the average Likert rating that the two compared variable statements received from each patient). When comparing the patient-physician rapport variable statements to the base accessibility variable statements, patients indicated the rapport variable statements to be more favored (x̄_d_ = 0.713, SD = 0.908, t = 8.606, *p* = 3.641E-14). Lastly, when comparing the confidence and rapport variable statements to one another, as well as those from the rapport and novel accessibility variable statements, there was no statistically significant difference between either compared group (x̄_d_ = 0.026, SD = 0.292, t = 0.968, *p* = 0.335 and x̄_d_ = 0.050, SD = 0.370, t = 1.481, *p* = 0.141, respectively). This suggests that patients believed these pairs of variable categories were approximately equally valuable when utilizing telemedicine through telephone appointments.

When analyzing the relationships between the general classes of variable categories (ie. patient-physician rapport vs confidence vs accessibility), t-tests of correlations revealed values as follows: confidence and rapport (*r* = 0.616, t = 10.790, *p* = 2.651E-19), confidence and novel accessibility (*r* = 0.525, t = 7.882, *p* = 1.789E-12), rapport and novel accessibility (*r* = 0.612, t = 10.643, *p* = 5.938E-19). However, confidence and base accessibility show a much lower correlation (*r* = 0.112, t = 1.238, *p* = 0.218), and rapport and base accessibility also had a relatively weak correlation (*r* = 0.182, *t* = 2.040, *p* = 0.044). These results suggest that although some variables from a given category were individually ranked as being more valued than others, no one class of variables is independently favored over another, which is to say that patients appreciate the presence of all listed variables in a telemedicine service.

Lastly, although not statistically significant, the cancellation/rescheduled surveys suggest a possible trend that patients may be more likely to cancel/delay their telemedicine appointment if hours of operation are inflexible (*n *= 33, x̄ = 1.970), if they are unable to pay for the encounter (*n* = 33, x̄ = 1.879), or if their provider is not easily accessible (*n* = 33, x̄ = 1.788). Further research is needed to confirm this correlation.

## Discussion

The study’s purpose was to evaluate patients’ views on novel accessibility variables of telemedicine and the attributes of telemedicine that resonate with patients. One of this modality’s key benefits is accessibility: patients do not need to physically enter a facility to receive medical care. Rather, with the appropriate resources, they can have remote encounters that meet their needs. However, our results suggest that accessibility is not solely about time and distance commuted, but also about flexible scheduling and how easily a medical professional can be reached. As such, the utilization of telemedicine seems to involve the low-hanging fruit principle, in which people tend to fulfill the tasks that are easy or convenient before attempting more difficult ones. Thus, the use of telemedicine services can result in patients being more consistent with their doctor visits and possibly, more compliant with their medical follow-ups.

Our findings also indicate that the more valued accessibility elements are those that give patients more control over appointment scheduling and the frequency with which telemedicine encounters/communications are available. This may be especially true for lower socioeconomic populations who may not have the ability to forgo a day of work or be able to afford childcare to attend a healthcare appointment [3]. Telemedicine allows patients to access medical care on their timetable, rather than having to coordinate their personal life around their medical condition. With such freedom, appointments would become less burdensome and patients would be more likely to attend them. In turn, this would help remedy previously identified traditional healthcare barriers, which include: patients being too busy to schedule/attend an appointment and a lack of patient access to treatments during regular hours of operation [20–23]. As such, it is advisable for telemedicine providers to survey their patient base to better understand their availability and thereby promote the service’s utility. However, healthcare systems should also consider patients’ digital literacy and whether they have the necessary resources to use the modality. Thus, further investigation is necessary to identify feasible methods for directly assisting disadvantaged patients.

With three of the top five patient-valued variables belonging to the confidence category, this suggests that confidence in a provider is an attribute patients are keen to possess through telemedicine care. In descending order of mean Likert ratings, these variables include services with 1) physicians and 2) staff who are perceived to be knowledgeable and 3) physicians and staff who are perceived as being trustworthy. These findings support those of previous studies [22, 24–26], which emphasize that medical personnel’s skillful demonstration of clinical knowledge is attractive when individuals select a particular healthcare institution. As such, telemedicine providers should be urged to invest time in establishing confidence between them and their patients.

One potential method involves physicians incorporating positive communication behaviors (e.g. providing opportunities for patient engagement, physician encouragement of patients, ensuring patients understand diagnoses, etc.) into their practice. Past studies found the use of such techniques to be directly correlated with patients’ perception of medical providers as competent, trustworthy, and kind [27, 28]. Each of these latter traits, as uncovered by McCroskey and Teven [29], ultimately contributes to credibility. With a heightened perception of physician credibility, Paulsel et al. [30] state that overall patient satisfaction with a service will increase, while also improving patients’ perception of their quality of care. Therefore, by taking time to apply positive communication techniques, physicians can demonstrate to their patients that they are well-qualified to tend to their medical needs. This is also the case with telemedicine visits, as our findings indicate that the same confidence-based variables are the most valuable for patients during their visits. In turn, positive communication techniques would help ameliorate previously identified in-person care hesitancy variables, which include fears of being: misdiagnosed, subjected to unnecessary tests, and prescribed unnecessary medications [20, 23, 24, 26]. Although physicians may feel as though they lack the time to incorporate such confidence- and rapport-building measures into their practice, Desjarlais-deKlerk and Wallace discovered that doing so would take roughly about the same time as it would if physicians strictly dispensed the required information to the patient and limited patients’ engagement in an interaction [31]. With some forms of telemedicine being inherently less personal (e.g. video or phone calls), failing to incorporate such measures can hinder medical providers’ ability to establish a confident and longitudinal relationship with their patients.

Yet, an additional benefit of granting patients the opportunity to engage during medical encounters is that it leads them to feel respected and as though their concerns have been acknowledged [32]. Meeting these latter two conditions was of great value for our study’s respondents, with the provision of care by physicians who are kind and helpful in addressing patients’ needs being the highest-rated variable of our questionnaire. According to Moore et al. [32], such rapport-building practices are invariably important to the patient-physician relationship. This is especially true with new patients, as an individual’s most recent experience with a medical provider influences whether they will continue pursuing future care with them. Furthermore, the favorable perception of their physician and their interactions tends to lead to increased levels of compliance as patients progress to the following steps of their care plan [23, 24]. Thus, to ensure the success of a telemedicine service (which is inherently less personal than in-person care), we reiterate the importance of exhibiting positive communication behaviors during all encounters.

In conclusion, our results suggest that rural patients favor the following general characteristics in telemedicine: flexible encounters and providers who strive to build trust and rapport. Future research to further understand why patients attend, cancel, or reschedule appointments using a free-response method is recommended, in addition to the assessment of patients’ technological fluency, to improve the efficacy and reach of telemedicine visits.

## Limitations

One study limitation includes participant response bias, as surveys were not administered anonymously and those conducted for the “completed appointment” category were done rather close in time to a patient’s appointment. Questionnaires could have also be written in a more neutral approach ensuring identical questions throughout versions. This study also does not assess the extent of patients’ previous experiences with telemedicine, which may have influenced their views. Additionally, our sample size for the cancellation/rescheduling survey was lacking to attain significant results. Finally, while our research primarily focused on patient views of telemedicine, it did not explore medical providers’ perspectives. Additional studies should be conducted to tease out their views on its accessibility benefits, the feasibility of meaningful rapport-building through such services, and whether telemedicine can be an effective adjunct to their field.

## Supplementary Information

Supplementary Material 1.

## Data Availability

The datasets used and/or analyzed during the current study are available from the corresponding author upon reasonable request.

## Abbreviations

ANOVA: An Analysis of Variance
LSD: The least significant difference
VVHC: Valley Vein Health Center
CVD: Chronic venous disease
IRB: Institutional Review Board
