# Supplementary material for: Variables influencing telemedicine utilization via telephone appointments among rural patients
Source: BMC Health Serv Res. 2025 Jan 2;25:1. doi: 10.1186/s12913-024-12122-5 (PMC11694379; doi:10.1186/s12913-024-12122-5)
Supplement: Supplementary file 1 — Supplementary Material 1. [file 12913_2024_12122_MOESM1_ESM.docx]

## Valley Vein Health Center Patient Survey

Patient Name: ______________________ Date: _______ Age: ______ Sex: M / F / Trans / Non-Binary

Spoken Language:_______________ Other Spoken Language(s):_______________

Race/Ethnicity: ___ Asian (Specify:_________) ___ American Indian/Alaska Native ___Indian/Pakistani/Punjabi ___ Pacific Islander ___ Hispanic/Latino

___ White (Not Hispanic/Latino) ___ Black/African American Other:________

Was this your first encounter with this clinician? Yes or No

If **no**, approximately how long have you been seeing us? 0-3 months 3-6 months 6-12 months 12+ months

What is the purpose of the visit? New Visit Followup Vein Treatment Post treatment followup Ultrasound Other:______

Type of visit: In person Telehealth

| **Please rate the following statements on how much they influenced your decision to choose this clinic for treatment. (5 for most and 1 for least)** | | | | | |
| --- | --- | --- | --- | --- | --- |
| The clinic follows COVID safety guidelines (plexiglass, air filters, social distancing, masks, cleanliness). | 5 | 4 | 3 | 2 | 1 |
| The treatment will help me get better. | 5 | 4 | 3 | 2 | 1 |
| They have great staff. | 5 | 4 | 3 | 2 | 1 |
| They have great doctors. | 5 | 4 | 3 | 2 | 1 |
| I trust the staff and doctors. | 5 | 4 | 3 | 2 | 1 |
| They do good treatments and take care of my medical condition. | 5 | 4 | 3 | 2 | 1 |
| They are kind and help me with what I need. | 5 | 4 | 3 | 2 | 1 |
| I can reach out to them when I have a question. | 5 | 4 | 3 | 2 | 1 |
| The staff are confident in what they say and do. | 5 | 4 | 3 | 2 | 1 |
| They called me frequently to explain things. | 5 | 4 | 3 | 2 | 1 |
| The staff speak to me in a language that I prefer. | 5 | 4 | 3 | 2 | 1 |
| They communicate well and give me clear information. | 5 | 4 | 3 | 2 | 1 |
| They discuss the treatment plan with me and listen to what I have to say. | 5 | 4 | 3 | 2 | 1 |
| They see me when I need or want. | 5 | 4 | 3 | 2 | 1 |
| Telehealth Follow Ups were available | 5 | 4 | 3 | 2 | 1 |
| Other people told me to come here. | 5 | 4 | 3 | 2 | 1 |
| It’s close to my home. | 5 | 4 | 3 | 2 | 1 |
| They helped me with getting a ride here. | 5 | 4 | 3 | 2 | 1 |
| They helped me when I couldn’t pay for a service. | 5 | 4 | 3 | 2 | 1 |
| How likely are you to return? | 5 | 4 | 3 | 2 | 1 |
| **The following statements are for telehealth ONLY. Please rate how much they influenced your decision to get treatment.** | | | | | |
| How many telehealth appointments have you had? | 5+ | 4 | 3 | 2 | 1 |
| Telehealth visits made you comfortable with proceeding with further treatment. | 5 | 4 | 3 | 2 | 1 |
| They keep my personal information safe. | 5 | 4 | 3 | 2 | 1 |
| They treated me as well as they would have in-person. | 5 | 4 | 3 | 2 | 1 |

##

## Valley Vein Health Center Patient Survey

Patient Name: ______________________ Date: _______ Age: ______ Sex: M / F / Trans / Non-Binary

Spoken Language:_______________ Other Spoken Language(s):_______________

Race/Ethnicity: ___ Asian (Specify:_________) ___ American Indian/Alaska Native ___Indian/Pakistani/Punjabi ___ Pacific Islander ___ Hispanic/Latino

___ White (Not Hispanic/Latino) ___ Black/African American Other:________

Was this your first encounter with this clinician? Yes or No

If **no**, approximately how long have you been seeing us? 0-3 months 3-6 months 6-12 months 12+ months

What is the purpose of the visit? New Visit Followup Vein Treatment Post treatment followup Ultrasound Other:______

Type of visit: In person Telehealth

| **Please rate the following statements on how much they influenced your cancellation or rescheduling of an appointment. (5 for most and 1 for least)** | | | | | |
| --- | --- | --- | --- | --- | --- |
| The facilities don’t have COVID safety guidelines (plexiglass, air filters, and social distancing). | 5 | 4 | 3 | 2 | 1 |
| The treatments won’t help me get better. | 5 | 4 | 3 | 2 | 1 |
| The staff is not helpful. | 5 | 4 | 3 | 2 | 1 |
| The doctors are not knowledgeable. | 5 | 4 | 3 | 2 | 1 |
| The staff are not trustworthy. | 5 | 4 | 3 | 2 | 1 |
| The treatments don’t improve my medical condition. | 5 | 4 | 3 | 2 | 1 |
| They were not understanding of my emotions or needs. | 5 | 4 | 3 | 2 | 1 |
| I can’t get a hold of them when I have a question. | 5 | 4 | 3 | 2 | 1 |
| The staff don’t convey confidence in what they say and do. | 5 | 4 | 3 | 2 | 1 |
| They don’t call me to explain what the medicine does or about the post treatment care. | 5 | 4 | 3 | 2 | 1 |
| The staff don’t speak to me in a language that I prefer. | 5 | 4 | 3 | 2 | 1 |
| They don’t communicate well or give me clear information. | 5 | 4 | 3 | 2 | 1 |
| They don’t discuss the treatment plan with me and are not attentive to what I have to say. | 5 | 4 | 3 | 2 | 1 |
| They can’t see me when I need or am available. | 5 | 4 | 3 | 2 | 1 |
| Telehealth Follow Ups were not available | 5 | 4 | 3 | 2 | 1 |
| I haven’t heard about this facility from anyone. | 5 | 4 | 3 | 2 | 1 |
| It’s far from my home. | 5 | 4 | 3 | 2 | 1 |
| I was unable to get a ride to my appointment. | 5 | 4 | 3 | 2 | 1 |
| I couldn’t pay for a service. | 5 | 4 | 3 | 2 | 1 |
| I thought the discomfort would go away on its own. | 5 | 4 | 3 | 2 | 1 |
| How likely are you to return? | 5 | 4 | 3 | 2 | 1 |
| **The following statements are for telehealth ONLY. Please rate how much they influenced your decision to cancel or reschedule further treatment.** | | | | | |
| How many telehealth appointments have you had? | 5+ | 4 | 3 | 2 | 1 |
| Telehealth visit didn’t make me feel comfortable moving forward with treatment. | 5 | 4 | 3 | 2 | 1 |
| My personal information didn’t feel safe. | 5 | 4 | 3 | 2 | 1 |
| I felt that an in-person appointment would have better met my needs. | 5 | 4 | 3 | 2 | 1 |
